# The Genetic Basis of Upland/Lowland Ecotype Divergence in Switchgrass (*Panicum virgatum)*

**DOI:** 10.1534/g3.116.032763

**Published:** 2016-09-08

**Authors:** Elizabeth R. Milano, David B. Lowry, Thomas E. Juenger

**Affiliations:** * Department of Integrative Biology, The University of Texas at Austin, Texas 78712; † Department of Plant Biology, Michigan State University, East Lansing, Michigan 48824

**Keywords:** genetic architecture, flowering time, local adaptation, QTL, trait syndromes

## Abstract

The evolution of locally adapted ecotypes is a common phenomenon that generates diversity within plant species. However, we know surprisingly little about the genetic mechanisms underlying the locally adapted traits involved in ecotype formation. The genetic architecture underlying locally adapted traits dictates how an organism will respond to environmental selection pressures, and has major implications for evolutionary ecology, conservation, and crop breeding. To understand the genetic architecture underlying the divergence of switchgrass (*Panicum virgatum*) ecotypes, we constructed a genetic mapping population through a four-way outbred cross between two northern upland and two southern lowland accessions. Trait segregation in this mapping population was largely consistent with multiple independent loci controlling the suite of traits that characterizes ecotype divergence. We assembled a joint linkage map using ddRADseq, and mapped quantitative trait loci (QTL) for traits that are divergent between ecotypes, including flowering time, plant size, physiological processes, and disease resistance. Overall, we found that most QTL had small to intermediate effects. While we identified colocalizing QTL for multiple traits, we did not find any large-effect QTL that clearly controlled multiple traits through pleiotropy or tight physical linkage. These results indicate that ecologically important traits in switchgrass have a complex genetic basis, and that similar loci may underlie divergence across the geographic range of the ecotypes.

Biological species are able to occupy a vast array of environmental conditions through adaptations driven by natural selection. Local adaptation is characterized by native populations consistently having greater fitness in their home habitat in comparison to foreign transplants from different habitats (Kawecki and Ebert 2004; Leimu and Fischer 2008; Hereford 2009). The majority of plant species have been found to be locally adapted based on empirical studies of fitness responses in reciprocal transplant studies (Leimu and Fischer 2008; Hereford 2009). Yet, we still know very little about the role that the genetic architecture underlying locally adapted traits plays in shaping how organisms respond to the environment in terms of their performance and fitness (Savolainen *et al.* 2013; Tiffin and Ross-Ibarra 2014). Further, local adaptation can be constrained or confounded by gene flow, lack of genetic variation, genetic drift, and the genetic architecture of traits (Kawecki and Ebert 2004; Hereford 2009).

Over time, local adaptation to different habitats can contribute to the formation of distinct ecotypes. The divergence of ecotypes can eventually lead to speciation through the evolution of ecological reproductive isolation (Ramsey *et al.* 2003; Kay 2006; Lowry *et al.* 2008; Glennon *et al.* 2012). Ecotypes generally differ in suites (*i.e.*, syndromes) of locally adapted traits from other populations (Turesson 1922a,b; Clausen 1951; Lowry 2012; Ravinet *et al.* 2016). These important trait correlations that characterize ecotypic divergence can result from pleiotropy or tight genetic linkage (Conner 2002; Wright *et al.* 2013; Mills *et al.* 2014). Alternatively, the traits that underlie syndromes that characterize ecotypic divergence could be under independent genetic control and be correlated as the result of linkage disequilibrium (LD) caused by strong correlational selection (Brodie *et al.* 1995). Understanding whether the suites of traits that characterize ecotype divergence are caused by pleiotropy/linkage or independent loci requires genetic crosses and quantitative trait analyses (Rogers and Bernatchez 2005; Hall *et al.* 2006; Lowry *et al.* 2015a,b).

In plants, ecotype formation is frequently driven by divergence in soil water availability across habitats, with ecotypes adapted to more mesic habitats being typically larger in size and flowering later than ecotypes from drier habits (Clausen and Hiesey 1958; Porter 1966; Latta *et al.* 2007; Lowry 2012). *Panicum virgatum* (switchgrass) is an ideal system for studying the evolutionary genetic basis of ecotype divergence. Switchgrass is a long-lived outcrossing C4 perennial grass native to a large region of central and eastern North America and extending south into Central America. It is a common species of the tallgrass prairie, utilized as a forage crop, and has been championed as a bioenergy feedstock (Casler *et al.* 2011; Casler 2012; Parrish *et al.* 2012). Switchgrass phenotypic diversity is characterized by two major ecotypes: “Upland” and “Lowland,” which are hypothesized to have descended from glacial refugia (McMillan 1959; Zhang *et al.* 2011b). Molecular studies support the distinctness of the two ecotypes by estimating deep dates of divergence between distinct upland and lowland haplotypes on the order of 0.7–1 million years ago (MYA) (Morris *et al.* 2011; Zhang *et al.* 2011b). Upland plants are typically found in drier soil conditions than lowland plants which typically reside in riparian habitats. The two ecotypes are easily distinguished by a suite of morphological differences, with lowland plants tending to have fewer and larger tillers, erect growth, a compact crown, blue-green waxy leaves, and late flowering time. Genetic variation in the cytoplasm is highly correlated with the divergence of the ecotypes (Morris *et al.* 2011; Zhang *et al.* 2011b). Although the species is polyploid (4× – 8×), recent full-sib linkage studies indicate tetraploid switchgrass maintains preferential pairing and disomic inheritance (Okada *et al.* 2010; Lu *et al.* 2013). Tetraploids of each ecotype are largely reproductively compatible (McLaughlin and Kszos 2005) and putative hybrids are found in regions of co-occurrence (Zhang *et al.* 2011a; Lowry *et al.* 2014). Porter (1966) showed that upland and lowland ecotypes are locally adapted to their respective habitats through a reciprocal transplant experiment. The upland ecotype was found to be more drought tolerant and have higher nitrogen demand than the lowland ecotype, which is more tolerant to flooding. Numerous other transplant and field trials have demonstrated phenotypic and physiological differences between the two ecotypes (Wullschleger *et al.* 1996; Casler and Vogel 2004; Cassida *et al.* 2005a,b; Barney *et al.* 2009; Yang *et al.* 2009; Cortese *et al.* 2010). For example, resistance to rust fungus infection has been found to be heritable and lowland populations are typically more resistant than upland populations (Eberhart and Newell 1959; Uppalapati *et al.* 2013).

In addition to ecotype divergence, adaptive phenotypic variation in switchgrass is driven by environmental variables that correlate with latitude. Classic research in switchgrass has demonstrated that phenological traits, including date of emergence, flowering time, and date of senescence are strongly correlated with latitude of origin in common garden experiments (McMillan 1959, 1965, 1967). Transplantation experiments and field trials have consistently demonstrated that moving genotypes north and south of their locations of origin results in a loss of fitness due to a suite of environmental factors (McMillan 1959, 1965, 1967; Porter 1966; Casler and Vogel 2004; Lowry *et al.* 2014).

Here, we developed a new outbred genetic mapping population to understand the genetic basis of adaptation to environmental factors that are divergent between northern upland and southern lowland ecotypes of switchgrass. The mapping population was formed through reciprocal crosses between four grandparents derived from different locations across the Great Plains of North America. Two of the grandparents are lowland accessions derived from the southern Great Plains, while the other two grandparents are upland accessions from the northern Great Plains (Figure 1). This balanced design, including upland/lowland cytoplasm, allows us to ask whether a shared set of loci are involved in adaptive divergence between southern lowland and northern upland populations or if different alleles and loci might be involved in reaching similar phenotypes in different local ecotype populations. We assessed this complexity through genetic mapping and characterizing allelic effects for quantitative trait loci (QTL) associated with ecotype divergence. Our results provide insight into the underlying genetic basis of adaptive ecotypic variation.

**Figure 1:**
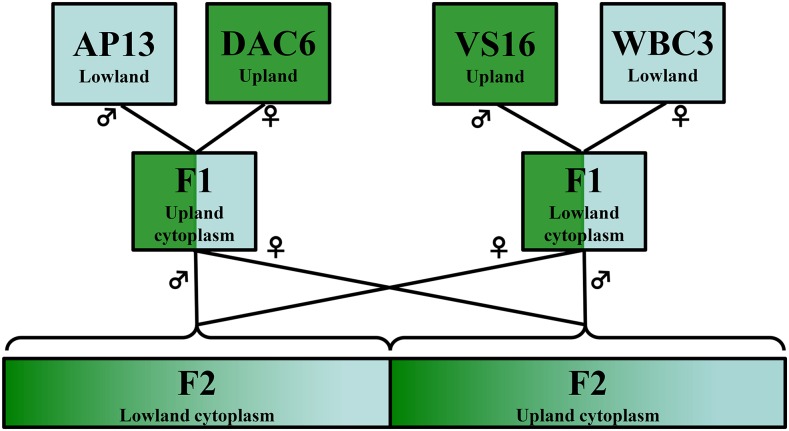
Diagram of four-way outbred reciprocal cross between two upland and two lowland ecotypes of *P. virgatum*.

## Materials and Methods

### Outbred mapping population

*P. virgatum* is an obligate outcrosser (Martínez-Reyna and Vogel 2002) and as such it is necessary to account for the fact that parental material will be genetically heterogeneous and thus generate many marker segregation types. We created a four-way phase-known (pseudotestcross) population to evaluate the genetic architecture of upland/lowland traits in switchgrass. In this scheme, two sets of grandparents [*lowland*_1_ × *upland*_2_ and *upland*_3_ × *lowland_4_*] were crossed to create F_1_ hybrids that were then reciprocally crossed to generate two large “outbred F_2_” populations (F_12♀_×F_34♂_, F_12♂_×F_34♀_) of 200 progeny each. We used four tetraploid grandparents in this design: Alamo (“AP13” genotype, the reference genome and southern Texas accession), West Bee Cave (“WBC3” genotype, a central Texas lowland ecotype), Summer (“VS16” genotype, a northern upland accession), and Dacotah (“DAC6” genotype, a northern upland accession). The Alamo and Summer grandparents functioned as pollen donors in crosses and therefore the two F_1_ hybrids and their subsequent outbred families differ in that they contain either lowland WBC3 or upland DAC6 cytotypes (Figure 1). Given disomic inheritance, each outbred family can segregate up to four unique alleles donated by the grandparents. Phase can be resolved from the multigenerational information. Sampling multiple grandparental alleles increases the possibility of evaluating informative QTL and inspection of the allelic effects of QTL may provide insight into genetic heterogeneity in ecotype divergence. For example, do QTL alleles from WBC and AP13 exhibit similar or differing additive effects relative to VS16 and DAC6 QTL alleles? Finally, the cytoplasmic segregation also allows for investigation into cytoplasmic and cytoplasmic by nuclear QTL interactions.

### Cultivation and phenotyping

Seed from the reciprocal F_1_ hybrid cross was germinated and initially planted in 4-inch pots in a greenhouse at the University of Texas at Austin. At the two true leaf stage they were transferred to 1-gallon pots using a potting mix of Promix (Premier Tech Horticulture, Riviére-du-Loup, Quebec, Canada) and Turface (Profile Products, Buffalo Grove, IL) in a ratio of 4:1. Plants were grown in a greenhouse under 16-hr days from January to June 2012. Each plant was scored for date to first flowering, from time of transfer to the 1-gallon pot to anthesis. Plant height was also quantified, as the total length of tallest tiller, on the day of first flower. Leaf tissue for genomic DNA extraction was collected and frozen in liquid N_2_. After flowering, the pots were moved from the greenhouse to an outdoor field nursery. At the end of the growing season, the plants were clonally divided by splitting the crown into generally equal halves. These two replicates were repotted in the same soil mixture using 1-gallon pots and maintained in the field nursery. One replicate of each of the mapping progeny genotypes and 20 replicates of each grandparent and F_1_ hybrid were transplanted into the field at the experimental garden site at the Brackenridge Field Labs in Austin, TX, in February 2014, while the other set of replicates was kept as backup tissue and maintained in the field nursery. The field planting was based on a randomized honeycomb design with 1.25 m interplant distances surrounded by a row of border plants to minimize edge effects. Weed barrier cloth (Sunbelt 3.2 oz.; Dewitt, Sikeston, MO) was used to aid in establishment and minimize plot maintenance. The common garden field site was located in lowland switchgrass habitat associated with Colorado River floodplain (30.284138**°**N, −97.781632**°**W), where the soil type is Yazoo sandy loam.

The following traits were measured during the 2014 growing season for field grown plants: date to first flowering, chlorophyll content (SPAD), specific leaf area (SLA), midday water potential (MD WP), total number of tillers, single tiller mass, number of leaves on five total tillers, height, and pathogen susceptibility. Date to first flowering (flowering date) is calculated as the number of days from emergence to first anthesis. We measured MD WP using a Scholander pressure chamber (PMS Instrument Company, Albany, OR) on a mature leaf. Water potential indicates the leaf water potential status, and when measured during the heat of the day, can indicate tolerance to water stress. Larger (more negative) water potential values indicate more water stress (Pérez-Harguindeguy *et al.* 2013). Relative chlorophyll content was estimated using a chlorophyll SPAD meter (Konica-Minolta SPAD 502; Konica-Minolta, Chiyoda, Tokyo, Japan). Three readings from a single mature leaf were taken and the mean value recorded. Relative chlorophyll content, or leaf “greenness,” was estimated by a corrected ratio of transmitted light with wavelengths of 940 and 650 nm (Markwell *et al.* 1995). SLA is the ratio of leaf area (square millimeter) to dry leaf mass (gram), the resulting value is a measure of leaf density (Pérez-Harguindeguy *et al.* 2013). We recorded the area of three mature leaves using a portable leaf area meter (LI-3000A; Li-Cor, Lincoln, NE) and recorded the mass of the same leaves after full desiccation. Larger values for the area-to-mass ratio indicate a thinner, less dense leaf. Total plant height, single tiller mass, total number of tillers, and number of leaves on five tillers were taken at the end of the growing season. Height was measured using a graded measuring rod and tiller mass was calculated by averaging the dry weight of five mature tillers stripped of leaves and inflorescences. We calculated phenotypic correlations among traits for the grandparent ecotypes and recombinant mapping progeny using the Spearman rank method on raw phenotype values. We used the Holm–Bonferroni correction for multiple testing (Holm 1979).

### Rust susceptibility

Infection by a fungal rust pathogen was observed in the mapping population in the potted plants in 2013. Plants were treated with a fungicide (Daconil GardenTech, Palatine, IL) to aid in vigorous establishment of clonal replicates, and all but 12 inches of above ground biomass was removed before dormancy. The plants were then planted in the field for the following 2014 growing season. Physiological phenotypes (see above, Cultivation and phenotyping) were measured on green, healthy leaves. At the end of the growing season, five mature tillers were harvested and manually stripped of their leaves. Each leaf was then scored for presence of the fungal pathogen using a qualitative four-point rating: 1, completely infected and brown in color, to 4, no evidence of pathogen and green in color (Supplemental Material, Figure S1). The total number of leaves on all five tillers was recorded as well. Each plant was then given a percentage score for each leaf category. We then used a principal component analysis for the four percentile scores to find the major axis of variation for the trait. The first principal component explained 86.4% of the variation and was largely associated with the percentage score of completely infected leaves. We therefore used the scores for the first principal component (Rust PC1) as a proxy for a pathogen-resistant phenotype.

### Genotyping

Fresh leaf tissue was collected in the greenhouse, immediately frozen in liquid N_2_, and stored at −80° in the spring of 2013. The equivalent of 100 mg of wet tissue was then used for genomic DNA extraction with the MasterPure Plant Leaf DNA Purification Kit (Epicentre, Madison, WI). The procedure was modified with an initial RNase A treatment and two subsequent ethanol washes. Final DNA was eluted in 30 μl TE buffer and quantified using the Broad Range spectrum kit of the Qubit 2.0 (Life Technologies, Carlsbad, CA). Each extraction yielded ∼30–200 ng/μl DNA.

Each plant was genotyped using a double-digest Restriction-site Associated DNA sequencing (ddRADseq) (Peterson *et al.* 2012) scheme. In brief, 300 ng of DNA was cut with *Eco*RI and *Sph*I enzymes. In-line barcodes were ligated onto the *Eco*RI cut site, fragments were size-selected on a Pippen Prep (Sage Science, Inc., Beverly, MA) at a 300 ± 30 bp range, then Illumina adaptors were ligated onto the *Sph*I cut site. This produced nine multiplexed libraries with 48 individuals each. Libraries were sequenced on an Illumina 2000 HiSeq (San Diego, CA) at the Genome Sequencing and Analysis Facility in Austin, TX. Raw read quality was evaluated with FastQC (v. 0.10.1). Library demultiplexing was performed in Stacks (v. 1.06, Catchen *et al.* 2011). Sequencing resulted in ∼1.6 million (mean = 1,626,329 ± 34,002.78 SE) raw reads per individual. Reads from each individual were then mapped to the *P. virgatum* v. 1.1 genome (DOE-JGI, http://phytozome.jgi.doe.gov) using BWA *mem* (BWA v. 0.7.9a, Li 2013; Li and Durbin 2010). Mapped reads were further processed in Stacks for genotype calling and allele designations. The two F_1_ hybrids were designated as parents in the Stacks ref_map.pl pipeline and 140,561 markers were included in the catalog of markers for scoring genotypes. Each individual was then genotyped at ∼38,000 (mean = 38,161.2 ± 670.0 SE) markers. The coverage for each marker was ∼40×. Due to missing data the dataset was filtered for markers present in at least 50% of the individuals. We also filtered for segregation distortion by testing for significant deviation from Mendelian expectations and removing all loci where *P* < 0.00005. This resulted in a high quality set of 1348 markers available for subsequent linkage analysis.

### Linkage map assembly and QTL analysis

Outbred mapping populations are often analyzed in a pseudotestcross approach, developing independent maternal and paternal linkage maps based on single dose markers that uniquely segregate in either parent (Haley *et al.* 1994). Here, we develop a joint linkage map based on the outbred full-sib family (CP) design using Joinmap (v. 4.1) and the multipoint maximum likelihood (ML) algorithm (van Ooijen 2011). This algorithm is unique in that it simultaneously estimates phase and recombination fraction and can utilize all possible segregation types in ordering markers and estimating marker interval distances. We first grouped markers into linkage groups (LGs) using a conservative logarithmic odds-ratio (LOD) of 10.0. We then used a simple regression algorithm to order markers on each group with the following settings: pairwise recombination frequency < 0.4, LOD > 3, with a Kosambi mapping function to calculate genetic distances. Markers that affected goodness of fit (mean chi-square > 3) were removed. We then used ML to order and estimate mapping distances for the edited groups. This produced a finalized map with 1281 markers.

We mapped QTL for phenotypic traits for the upland/lowland outbred mapping population using a stepwise multiple-QTL model fitting method as implemented in R/qtl (Broman *et al.* 2003; Manichaikul *et al.* 2009). A generalized linear model was used to correct for the effects of potting cohort for traits measured in the greenhouse. Field traits did not need a cohort correction and were quantile normalized to create a uniform set of normally distributed traits. All QTL scans were performed using a normal model and Haley-Knott regression based on a dense 2 cM grid of pseudomarkers generated using the calc.genoprob function. We calculated LOD penalties for main effects and interactions for each trait through 1000 permutations of the scantwo function at an α of 0.05. We conducted a forward/backward stepwise search for models with a maximum of 10 QTL that optimized the penalized LOD score criterion. We calculated the 1.5 LOD drop interval of QTL in the best-fit models and the percentage variance explained for each QTL based on the final best-fit models using the fitqtl function. We designated cytoplasm (cross direction) as an additive and interactive covariate for the scantwo penalty calculation. We also designated cytoplasm as an additive covariate for the stepwise model fitting and performed a *post hoc* nuclear-by-cytoplasm interaction test. Computation was performed on the Lonestar cluster at the Texas Advanced Computing Center at the University of Texas at Austin (www.tacc.utexas.edu) using custom scripts (github.com/ermilano/4way).

### Data availability

Raw sequence reads are available at the NCBI SRA, accession number: SAMN05609456 (http://www.ncbi.nlm.nih.gov/biosample/5609456). File S1 contains barcode information. File S2 is an R/qtl file that contains the joint genetic map and genotype and phenotype data for each individual in the QTL analysis. File S3 contains metadata for File S2.

## Results

### Trait variation, divergence, and correlations

Upland and lowland ecotypes of switchgrass are characterized by divergence in a variety of traits including differences in flowering time, growth architecture, physiological characteristics, and disease susceptibility (McMillan 1964, 1965, 1967; Casler *et al.* 2011; Uppalapati *et al.* 2013). Most traits we measured showed the expected trait differentiation between the upland and lowland ecotypes based on the mean values of each of the grandparents, including height (lowland > upland), tiller mass (lowland > upland), SLA (upland > lowland), rust resistance (lowland > upland), flowering time (lowland > upland), and leaf number (lowland > upland) (Figure S2). SPAD and MD WP did not differ between upland and lowland ecotypes, and tiller count was in the opposite direction of the pattern anticipated; lowland genotypes developed more tillers than upland genotypes during this establishment year. The phenotypic distribution of the traits in the F_1_ individuals and the F_2_ mapping progeny was generally unimodal and exhibited limited transgressive segregation (Figure S2). For example, flowering time in the F_2_ progeny ranged from 5 to 78 d, surpassing the maximum grandparental ecotype value of 64 d from lowland AP13. We did not observe a significant effect of cytoplasm on any of the measured phenotypes (ANOVA *P* > 0.05 for all trait comparisons across cytoplasmic backgrounds, data not shown).

**Figure 2:**
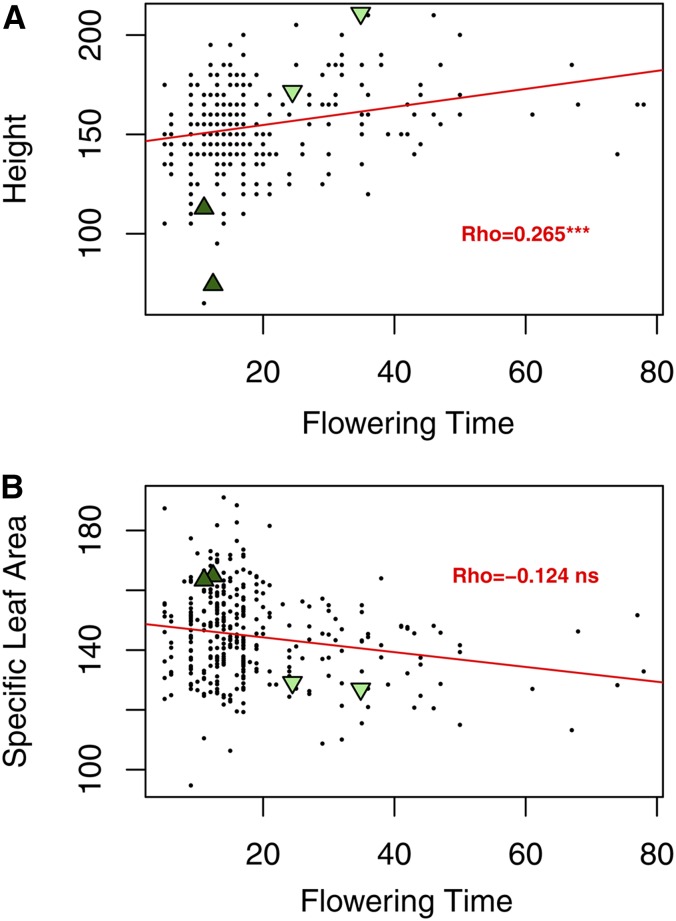
Sample scatterplots of trait correlations, (A) flowering time and height, and (B) flowering time and specific leaf area, in the F_2_ population with linear regression line (red) and averaged trait values for the upland (dark green triangles) and lowland (light green triangles) grandparent replicate plants. ***, *p* < 0.001; ns, not significant.

Ecotypes are comprised of suites of divergent phenotypes associated with adaptation to contrasting habitats. In the grandparents, we expected traits to be correlated with each other as a result of ecotypic divergence. For example, there is a positive relationship between flowering date and height due to short stature and early flowering date of the upland ecotype compared to the tall stature and later flowering date of the lowland ecotype. All correlations were positive except with SLA, where the correlations were strongly negative. This is expected as the smaller, thinner upland leaves have a larger leaf area-to-mass ratio. Genetic recombination can reduce the number of pairwise trait covariances if the traits are largely polygenic and not controlled by a few pleiotropic loci of major effect (Conner 2002). We calculated a correlation coefficient for all pairwise trait combinations in the progeny and found 14 significant correlations out of 36 total (Table 1). We found that some traits remained highly correlated after recombination, for example, flowering date and height measured in the field (Figure 2A), whereas other trait pairs do not have a significant relationship, for example flower date and SLA (Figure 2B).

**Table 1:** Spearman’s rank correlation (ρ) for phenotypic traits measured on the mapping population in the field

	Flower Date	Height	Tiller Mass	Tiller Number	Leaf Number	MD WP	SPAD	SLA
Height	0.265***							
Tiller Mass	0.232***	0.639***						
Tiller Number	0.157 ns	0.341***	0.165*					
Leaf Number	0.066 ns	0.314***	0.408***	0.066 ns				
MD WP	0.216 **	0.09 ns	0.09 ns	0.064 ns	0.003 ns			
SPAD	0.101 ns	0.14 ns	0.151 ns	0.1 ns	−0.041 ns	0.084 ns		
SLA	−0.124 ns	−0.22***	−0.271***	0.065 ns	−0.199**	−0.262***	−0.04 ns	
Rust PC1	0.149 ns	0.071 ns	−0.009 ns	−0.232***	−0.087 ns	0.221***	0.053 ns	−0.147 ns

* *P* < 0.05, ** *P* < 0.01, *** *P* < 0.001; ns, not significant. *P*-values corrected for multiple tests using the Holm–Bonferroni method. MD WP, midday water potential; SPAD, chlorophyll content; SLA, specific leaf area; Rust PC1, pathogen resistance.

### Linkage map

**Figure 3:**
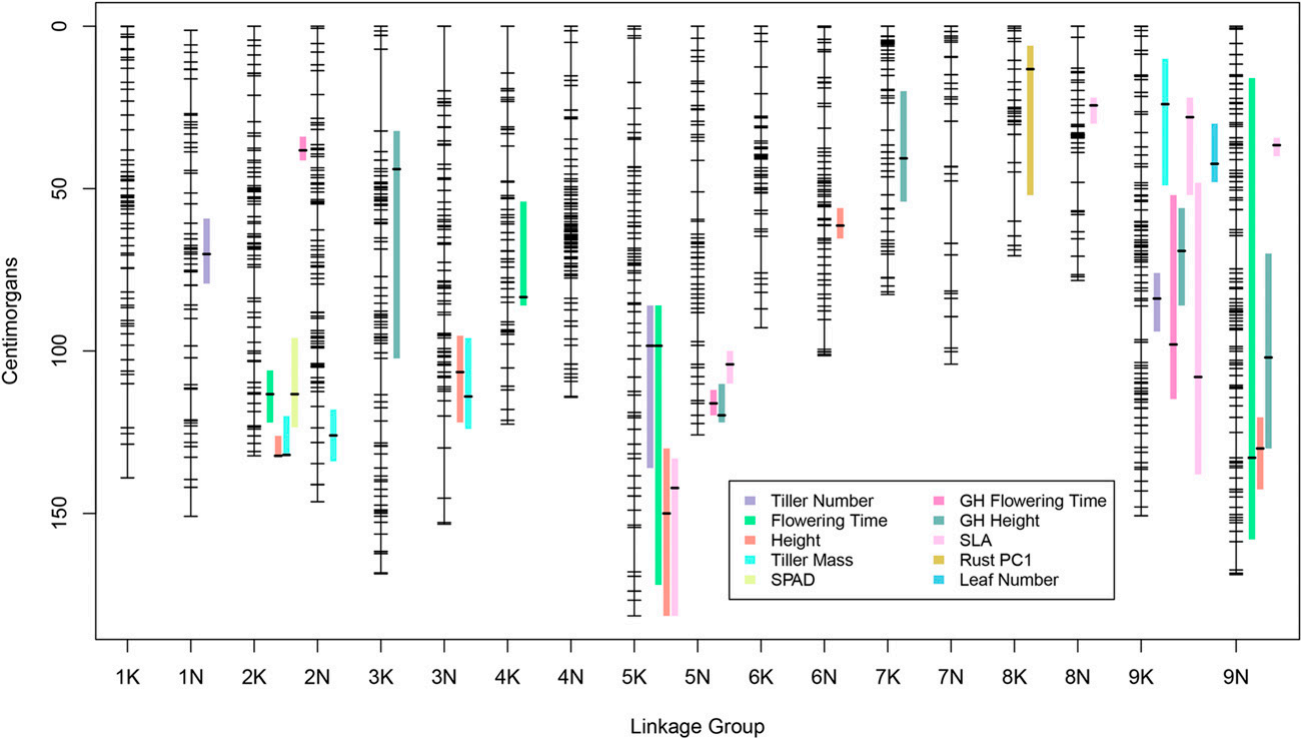
Genetic linkage map for *P. virgatum* with QTL and 1.5 LOD drop confidence intervals mapped to the right of their respective linkage groups. GH, greenhouse trait.

We successfully ordered 1281 ddRADseq markers on 18 distinct LGs, corresponding to the nine chromosomes of the tetraploid switchgrass N and K subgenomes (Figure 3). The total map distance is 2288.7 cM, with an average intermarker map distance of 1.8 cM (±0.55 SE). This map length is comparable to the Kanlow-by-Alamo pseudotestcross map at 2200.4 cM (Lowry *et al.* 2015b), but larger than separate male and female F_1_ maps [Alamo male (1515 cM) and Kanlow female (1935 cM), Okada *et al.* 2010; Alamo female (1733 cM) and Summer male (1508 cM) maps Serba *et al.* 2013]. One third of the ddRADseq markers mapped to unanchored contigs in the *P. virgatum* reference genome, and we were able to place these markers onto LGs. There are several marker segregation types that result from an outbred cross depending on the heterozygosity in each of four grandparents (Wu *et al.* 2010). For example, four unique grandparental alleles (<abxcd>) result in a fully informative marker for both linkage and QTL mapping. However, loci with fewer than four unique alleles result in partially informative markers (*e.g.*, <efxeg>). We found 798 partially informative biallelic markers, 450 partially informative triallelic markers, and 33 fully informative markers. The fully informative markers uniquely identify the contributed grandparental chromosomes and were used to phase each LG for analysis of allelic effects.

### QTL

Overall, we identified 33 QTL and three epistatic interactions across 11 traits, using stepwise model selection (Figure 3). The largest additive effect QTL was located on LG 5N at 116.12 cM, with 19.3% variance explained [percent of phenotypic variance explained by QTL (PVE)] for flowering time in the greenhouse. The largest additive effect QTL in the field were for SLA on LG 9N at 36.63 cM (13.46 PVE), and plant height on LG 2K at 132.27 cM (13.34 PVE) (Table 2). The only trait lacking significant QTL was MD WP. Notably, we found a total of seven QTL on LG 9K. Over two thirds of the QTL peaks were found on one third of the LGs, with 72.7% of the QTL localized to the tetraploid homoeologue pairs 2, 5, and 9. We did not detect any significant (*P* > 0.05) cytoplasmic or nuclear-by-cytoplasmic interactions.

**Table 2:** Significant QTL for traits measured in the field and in the greenhouse

Phenotype	Linkage Group	Position (cM)	1.5 LOD C.I. (cM)	LOD	PVE	PDE	Effect Direction
Flowering Date	2K	113.28	106–122	9.13	9.1	74.09	+
Flowering Date	4K	83.41	54–86	8.77	8.72	70.99	−
Flowering Date	5K	98.4	86–172	5.32	5.17	42.09	+
Flowering Date	9N	132.87	16–158	5.09	4.94	40.22	−
Height*^a^*	2K	132.27	126.14–132.27	14.01	13.34	39.08	+
Height	3N	106.5	95.3–122	5.68	5.13	15.03	−
Height	5K	150	130–181.56	4.39	3.93	11.51	+
Height*^a^*	6N	61.39	56–65.35	13.32	12.63	37	−
Height	9N	130	120.45–142.6	5.81	5.25	15.38	−
Tiller Mass	2K	132	120–132.27	6.35	6.48	14.6	−
Tiller Mass	9K	24	10–49	6.55	6.69	15.07	+
Tiller Mass	2N	126	118–134	6.85	7.01	15.8	+
Tiller Mass	3N	114	96–124	7.45	7.65	17.24	+
Tiller Number	1N	70.15	59.25–79.25	7.1	8.19	85.8	+
Tiller Number	5K	98.4	86–136	4.46	5.06	53.01	+
Tiller Number	9K	83.86	76–94	4.43	5.02	52.59	−
Leaf Number	9K	42.37	30–48	6.36	8.25	80.82	+
SPAD	2K	113.28	96–123.5	5.67	7.43	97.94	−
Specific Leaf Area	5K	142.18	133.1–181.56	4.57	3.68	−19.66	−
Specific Leaf Area	5N	104.11	100–110	7.4	6.06	−32.37	+
Specific Leaf Area*^a^*	8N	24.38	22–30	13.87	11.85	−63.31	+
Specific Leaf Area*^a^*	9N	36.63	34.39–40	15.59	13.46	−71.91	+
Specific Leaf Area	9K	28	22–51.984	8.75	7.22	−38.57	+
Specific Leaf Area	9K	108	48.21–138	4.64	3.73	−19.93	+
Rust PC1	8K	13.21	6–52	4.86	6.22	25.91	+
GH Flowering Date*^a^*	2K	38.23	34–41.32	12	11.9	NA	−
GH Flowering Date*^a^*	5N	116.12	112–119.8	18.67	19.31	NA	+
GH Flowering Date	9K	98	52–114.79	4.31	4.08	NA	+
GH Height	3K	44	32.24–102.27	6.04	5.47	NA	+
GH Height	5N	119.8	110.13–122	6.08	5.5	NA	+
GH Height	7K	40.69	20–54	5.06	4.54	NA	+
GH Height	9N	102	70–130	4.79	4.3	NA	+
GH Height	9K	69.18	56–86	5.41	4.88	NA	+

Each row represents a single QTL peak. LOD, logarithm of odds; C.I., confidence interval; PVE, percentage of phenotypic variance explained by QTL; PDE, percentage of parental divergence explained by QTL; +, allelic effect consistent with ecotype divergence; −, allelic effect opposite of ecotype expectation; GH, greenhouse trait.

a Epistatic interaction between QTL within trait.

We found six QTL and one epistatic interaction for SLA, the largest number of QTL per trait in our study. Together, these QTL totaled 57.23 PVE for that trait. We also found five QTL for both measurements of height, in the greenhouse and in the field. Interestingly, there was only one region, on LG 9N from 120 to 130 cM, where QTL for both height measurements colocalized. We detected three and four QTL for flowering time in the greenhouse and in the field respectively. However, while each had a QTL on LG 2K, the confidence intervals did not overlap. We discovered three pairwise epistatic interactions that explained a moderate amount of variance relative to the additive effects. These interactions were found for height (8.12 PVE), SLA (9.43 PVE), and greenhouse flowering time (11.22 PVE). We found significant rank changes in the allelic effects depending on genetic background within each case but there was no consistent pattern across the three separate interactions. Three traits (leaf number, SPAD, and rust resistance) yielded a single QTL but each QTL was <10 PVE, suggesting there may be many undetected loci controlling these traits. However, we acknowledge the limitation of using single replicates of each genotype. Power to detect small effect loci can be increased with more replication, especially for low heritability traits.

### Allelic effects

**Figure 4:**
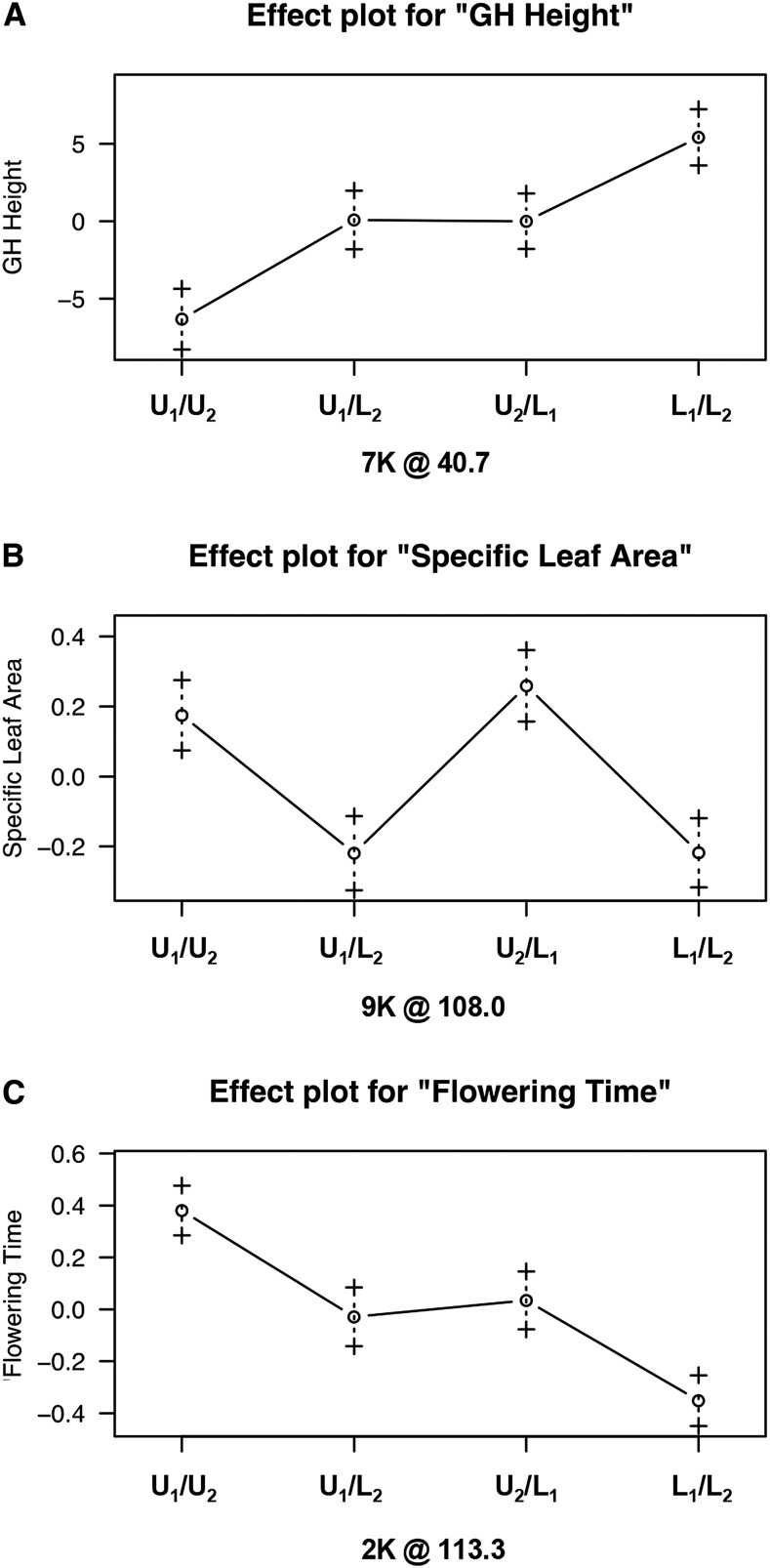
Selected allelic effects plots to illustrate (A) fixed ecotype effects, (B) polymorphic ecotype effects, and (C) fixed ecotypic effect in the opposite direction of expected ecotype divergence. The *x*-axis indicates genotype. X-axis subtitle indicates LG and marker position of the specific locus. GH, greenhouse traits; L_1_, AP13; L_2_, WBC3; U_1_, DAC6; U_2_, VS16.

The use of an outbred mapping population affords a more detailed evaluation of QTL allelic effects than traditional inbred line crosses given the contribution of potentially four grandparental QTL alleles in the F_2_ progeny. After phasing, the allelic affects at QTL can be evaluated based on the four possible recombinant progeny, including upland homozygotes (VS16/DAC6), lowland homozygotes (AP13/WBC3), and the two upland/lowland hybrid genotypes (VS16/AP13 and DAC6/WBC3). Inspection of the additive effects can therefore allow some interpretation of the distribution of functional alleles within and between ecotypes and the genetic mechanism associated with each trait. For example, the functional alleles can be consistent between ecotypes where unique upland and lowland genotypes contribute the same functional alleles as determined by the magnitude and direction of their additive effects (Figure 4A). Or functional alleles can be polymorphic within ecotypes, where individual genotypes contribute unique functional alleles to progeny irrespective of ecotype; a primary indication of this pattern would be the observation of strongly differing phenotypes in the two unique upland/lowland heterozygotes at a particular QTL (Figure 4B). We can also evaluate whether the direction of the allelic effects is the pattern expected given the overall phenotypic divergence between upland/lowland ecotypes. We found 10 of the 33 QTL had additive effects in the opposite direction of that expected for divergence between upland and lowland ecotypes (Table 2). For example, lowland ecotypes consistently flower later than uplands, but the lowland alleles for flowering date on LG 2K resulted in an earlier flowering time than upland alleles (Figure 4C). All five QTL on LG 2K, three out of four QTL on LG 5K, and one each on LGs 9N and 6N exhibited allelic effects in the opposite of expected upland/lowland ecotypic divergence.

## Discussion

To understand the genetic architecture of divergence between upland and lowland ecotypes of switchgrass, we assembled a genetic linkage map and conducted QTL mapping for a reciprocal four-way cross. The results of QTL mapping allowed us to then characterize functional allelic effects for traits associated with ecotype divergence. Segregation of phenotypic variation within the mapping population suggests that the suite of traits characterizing ecotypic divergence is the result of complex genetic architecture that involves limited evidence for any large effect or pleiotropic loci. QTL mapping also supported this conclusion, with loci controlling trait divergence distributed throughout the genome. We identified multiple QTL with additive effects that are consistent with patterns of ecotypic divergence. This result suggests that some of the same loci may be involved in upland/lowland ecotype divergence across the geographic range of switchgrass. Overall, our results provide a better understanding of the genetic architecture underlying ecotype divergence and set the stage for improvement of regionally adapted cultivars of switchgrass.

### The evolution of ecotypes

Ecotype divergence is typically characterized by a suite of trait differences that are correlated with environmental conditions that compose habitats (Clausen and Hiesey 1958; Lowry 2012). Ecologists often focus on documenting similar suites of trait that are correlated across genotypes and species, which they refer to as trait syndromes (Tjoelker *et al.* 2005; Possen *et al.* 2015). However, without genetic approaches it is not possible to determine whether trait syndromes are driven by pleiotropy/linkage or independent loci with allelic variation structured across ecotypes. Allelic variation can become structured between ecotypes through a buildup of LD across physically unlinked loci as a result of strong divergent and correlational selection. In a scenario where there is little to no gene flow between ecotypes, and selection is acting on suites of beneficial traits, we expect LD to build between loci associated with all traits that contribute to local adaptation. We also expect to see a significant reduction in LD and subsequent correlational structure following gene flow between ecotypes in nature, or through the controlled crosses in our mapping study. In our mapping population, we found that 11 of the trait correlations that were significant in the grandparents were no longer significant in the F_2_ generation, suggesting that those trait correlations were the result of LD due to population structure between the ecotypes.

While much of the trait syndrome that characterizes the upland and lowland ecotype divergence was due to independent loci, we also found evidence of tight physical linkage and/or pleiotropy. Tight physical linkage and pleiotropy can both facilitate and constrain adaptation, depending on the direction of additive effects on different traits. If allelic effects across traits are in the direction consistent with local adaptation, then tight linkage and pleiotropy can facilitate ecotype formation (Mills *et al.* 2014; Schwander *et al.* 2014). However, if allelic effects on traits are in the opposite direction of local adaptation, then the evolution of these traits will be constrained due to genetic trade-offs (Kawecki and Ebert 2004; Savolainen *et al.* 2013; Tiffin and Ross-Ibarra 2014). In our study, we found that tiller mass and SLA, as well as height and SLA, were significantly correlated in both the grandparents and the mapping progeny. The raw correlation value was negative but this is the expected direction for ecotype divergence. Tiller mass and SLA shared overlapping QTL confidence intervals on LG 9K, and both had effects in line with ecotypic divergence. Thus, physical linkage or pleiotropy may facilitate adaptive evolution of these traits. In contrast, height and SLA on LG 5K differed in direction of allelic effects. This genetic architecture could constrain response to selection along the axis of ecotype divergence.

Functional genetic variation is also often correlated with latitude, which reflects adaptation to environmental gradients that track latitude (Langlet 1971; Adrion *et al.* 2015). In switchgrass, much of the functional genetic variation has been shown to be strongly correlated with latitude (Casler and Vogel 2004; Casler *et al.* 2007; Lowry *et al.* 2014). The design of our study confounds the effects of ecotype and latitude because we only included northern upland and southern lowland individuals as grandparents in our crosses. While the lowland ecotype does not occur in northern regions, the two ecotypes overlap each other over a great portion of their range, with upland populations occurring at least as far south as Texas (Lowry *et al.* 2014). To fully partition latitudinal and ecotype effects, mapping populations would need to be made between southern lowland and southern upland plants.

### QTL colocalization

Our QTL findings are consistent with other *P. virgatum* studies, and we provide possible evidence for genomic regions across the *Panicum* complex, with consistent and conserved effects on traits. We found several regions in the genome where QTL confidence intervals overlapped across traits, mainly on tetraploid homoeologous pairs 2, 5, and 9. LG 2K is particularly interesting because all of the QTL effects were in the opposite direction than expected, based on patterns of ecotypic divergence. This may be due to several factors that we address below (see *Allelic effects)*. We found seven QTL that localized to LG 9K. This is consistent with previous mapping efforts of similar traits by Serba *et al.* (2014) and Lowry *et al.* (2015a,b). Both studies found major biomass QTL on LG 9K.

Another interesting comparison is to QTL recently identified in the diploid relative of switchgrass, *P. hallii*. The reference genome for switchgrass is anchored on the *P. hallii* genome and switchgrass and *P. hallii* are estimated to have diverged 5.3 MYA (Zhang *et al.* 2011b). *P. hallii* has two genomic hotspots of ecotype divergence, where QTL for many traits associated with ecotype divergence colocalize on LG 3 and LG 5 (Lowry *et al.* 2015a,b). LG 5 is particularly interesting because QTL for tiller number, flowering time, and height colocalized to the lower arm of LG 5 in *P. hallii* and LG 5K in switchgrass. Additionally, the lower arm for switchgrass LG 5N contained greenhouse-specific QTL for flowering time and height.

The earliest polyploidization in switchgrass was estimated to be roughly coincident with ecotype divergence occurring ∼1.2 MYA (Zhang *et al.* 2011a). We found that SLA had two QTL on the lower arms of LG 5K and LG 5N and the upper arms of LG 9K and LG 9N, suggesting loci may have retained function across subgenomes rather than diverged or degenerated as a result of genome duplication.

### Allelic effects

Since upland and lowland ecotypes are estimated to have diverged 0.7–1 MYA (Morris *et al.* 2011; Zhang *et al.* 2011b) during the Pleistocene, we might expect the majority of functional alleles to be fixed between ecotypes because of putatively strong and consistent divergent selection pressures. However, if there were very low levels of gene flow across the geographic range of switchgrass populations, we might expect functional alleles to be unique and varied among populations of each ecotype. This is consistent with a model of ecotype divergence stemming from ancient glacial refugia followed by adaptive radiations and genetic bottlenecks with each glacial cycle (Zhang *et al.* 2011b), and occasional gene flow across ecotype boundaries. In this model, ecotypes were originally formed from strongly selected and possibly small populations. However, there have been several glacial episodes since the estimated time of ecotype divergence, and with that, additional population bottlenecks and subsequent bouts of range shifts and changes in environmental selection pressures. Overall, we found that half of the QTL exhibited a pattern suggestive of fixed differences between ecotypes, supporting a model of considerable allelic heterogeneity both between and within upland and lowland ecotypes. Additional crosses and genetic mapping populations sampling a larger geographical distribution of both uplands and lowlands will be valuable for further insight into the evolution of switchgrass ecotypes.

Another important aspect of characterizing QTL is the direction of allelic effects. Even though QTL studies are known to both overestimate and underestimate effect size of QTL (Beavis 1998), there is very little bias in detecting direction of effect. We found that 30% of our QTL had allelic effects in the opposite direction than expected for upland and lowland ecotype divergence. Here we offer some possible explanations. In addition to the directional selection imposed by heterogeneous habitats, populations may experience stabilizing selection, or maintain polymorphisms in populations in the overlapping ranges that experience higher levels of gene flow. In the case of flowering time and height, two traits that are strongly divergent between the ecotypes, we found half of the allelic effects were in the opposite direction of expected ecotype divergence. One possibility in this case is that the trait is composed primarily of many very small effect loci, with allelic effects in the expected direction that are beyond the level of detection of this study. We found one particular region on the lower arm of LG 2K where all effects were in the opposite direction of ecotypic expectation. This may be the result of a chance fixation of a maladaptive chromosome block due to genetic drift in a population bottleneck (Orr 1998), or from a recent selective sweep for a trait we did not measure.

### Pathogen resistance

Rust infections of switchgrass pose major challenges to biofuel production as they can reduce ethanol yields up to 55% (Sykes *et al.* 2015). It is well known that the lowland switchgrass ecotype is more resistant to rust than the upland ecotype (Hopkins *et al.* 1995; Uppalapati *et al.* 2013). Fungal pathogens are generally moisture- and temperature-sensitive and often require high relative humidity for infection and sporulation (Harvell *et al.* 2002). Thus, a higher pathogen load could have driven greater resistance in southern lowland populations. Whatever the cause, little is known about the genetic architecture of this resistance. The natural infection that occurred during our experiment allowed us to screen for resistance QTL. We detected one resistance allele from the lowland WBC3 genotype using our coarse scale phenotype method. This lays the groundwork for future pathogen resistance mapping efforts in switchgrass that can be used to develop a panel of resistance alleles. Marker-assisted breeding programs can quickly and efficiently develop cultivars for different climactic regions for traits not amenable to transgenic manipulation and genetic engineering. Locating causal genes within QTL is costly, time intensive, and not useful if the phenotypic effect of each QTL is small. Yet it would be possible to introduce resistance alleles into the upland genetic background through several rounds of targeted marker-assisted breeding (Vogel and Jung 2001).

### Conclusions

Overall, our study has established a better understanding of the genetic architecture of ecotype divergence in switchgrass. In addition to a conceptual framework for the genetics of locally adapted ecotypes, we provide a starting point for marker-assisted selection of desired traits in a lignocellulosic bioenergy feedstock. Understanding and exploiting locally adapted traits in different genotypes will allow us to efficiently grow switchgrass in many different geographical regions economically, and with minimal input and ecological impact. Since there appears to be only limited pleiotropy underlying the divergence of upland and lowland ecotypes, it should be possible to improve many traits through breeding without incurring major phenotypic costs in other traits.

Going forward, we plan to utilize the four-way mapping population to better understand the genetic architecture of local adaptation across different latitudes. Clonal replicates of this mapping population are currently being planted in common gardens across the latitudinal gradient of the North American Great Plains to address clinal variation and genotype-by-environment interactions in *P. virgatum*. These complex interactions, in addition to ecotype divergence, are important considerations in habitat restoration, plant breeding, and accuracy of agronomic modeling.

## 

## Supplementary Material

Supplemental Material
