# Supplementary material for: The Genetic Basis of Upland/Lowland Ecotype Divergence in Switchgrass (Panicum virgatum)
Source: G3 (Bethesda). 2016 Sep 8;6(11):3561–70. doi: 10.1534/g3.116.032763 (PMC5100855; doi:10.1534/g3.116.032763)
Supplement: Supplemental Material [file supp_6_11_3561__index.html]

The Genetic Basis of Upland/Lowland Ecotype Divergence in Switchgrass (Panicum virgatum) — Supplemental Material 

# The Genetic Basis of Upland/Lowland Ecotype Divergence in Switchgrass (*Panicum virgatum)*

## Supplemental Material for Milano, Lowry, and Juenger, 2016

**Files in this Data Supplement:**

- Figure S1 - Photo of fungal pathogen infection. (.pdf, 69 KB)
- Figure S2 - Histograms of raw phenotypic values in the mapping population for all traits measured in the field. (.pdf, 189 KB)
- File S1 - Index and inline barcode sequences used to demultiplex raw sequence reads. (.txt, 8 KB)
- File S2 - R/qtl file containing the joint genetic map and genotype and phenotype data for each individual in the QTL analysis. (.csv, 1 MB)
- File S3 - Metadata for File S2 including phenotype descriptions and updated linkage group information. (.xlsx, 33 KB)
