# Supplementary material for: The Genetic Basis of Upland/Lowland Ecotype Divergence in Switchgrass (Panicum virgatum)
Source: G3 (Bethesda). 2016 Sep 8;6(11):3561–70. doi: 10.1534/g3.116.032763 (PMC5100855; doi:10.1534/g3.116.032763)
Supplement: Supplemental Material [file supp_g3.116.032763_FigureS1.pdf]

Figure S1

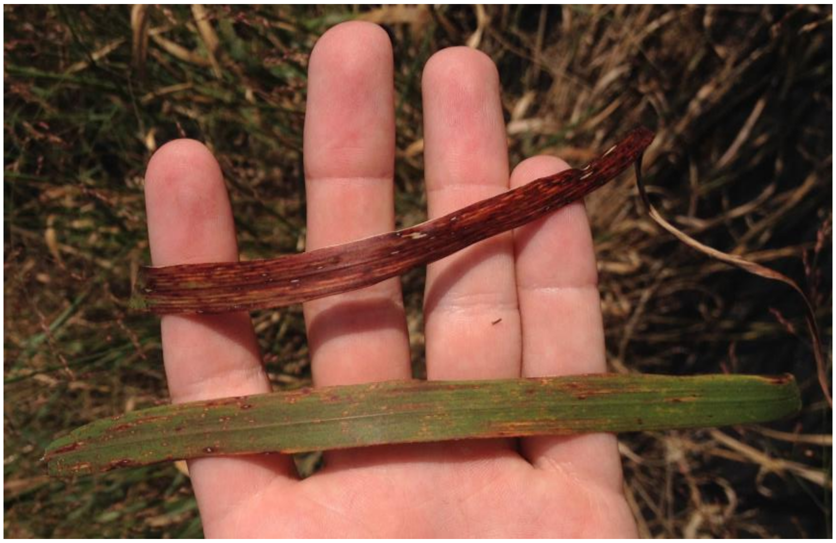

Figure S1

Photo of fungal pathogen infection. Fully infected leaf with a score of 1 on top, partially infected leaf with a score of 3 on bottom.
