# Supplementary material for: The Genetic Basis of Upland/Lowland Ecotype Divergence in Switchgrass (Panicum virgatum)
Source: G3 (Bethesda). 2016 Sep 8;6(11):3561–70. doi: 10.1534/g3.116.032763 (PMC5100855; doi:10.1534/g3.116.032763)
Supplement: Supplemental Material [file supp_g3.116.032763_FigureS2.pdf]

## Figure S2

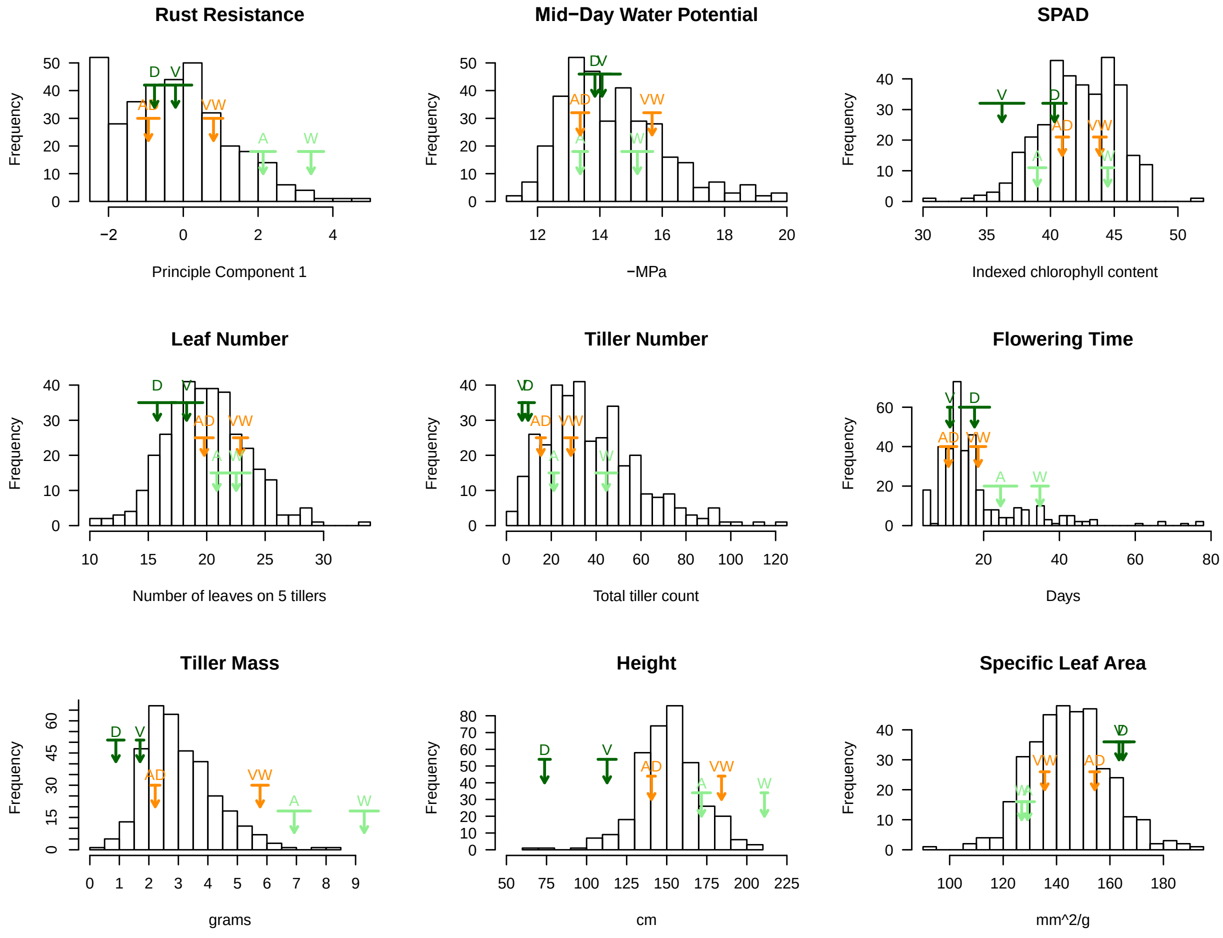


## Figure S2

Histograms of raw phenotypic values in the mapping population for all traits measured in the field, with mean values for each of the 2 original upland (dark green), lowland (light green) and $F_1$ hybrid (orange) individuals indicated by a vertical arrow, and standard error indicated by a horizontal bar. A- AP13; D- DAC6; V-VS16; W- WBC3; AD and VW- F1 parents.
